# Protective effects of Salidroside on cardiac function in mice with myocardial infarction

**DOI:** 10.1038/s41598-019-54713-x

**Published:** 2019-12-02

**Authors:** Pengsheng Chen, Jia Liu, Hongyun Ruan, Miaomiao Zhang, Peng Wu, Du Yimei, Bing Han

**Affiliations:** 1 Department of Cardiology, XuZhou Central Hospital, Xuzhou Clinical School of Nanjing Medical University, XuZhou Institute of Cardiovascular disease, Xuzhou, 221009 China; 2 Department of Clinical laboratory, XuZhou Central Hospital, Xuzhou Clinical School of Nanjing Medical University, Xuzhou, 221009 China; 3 Department of Cardiology, The First Affiliated Hospital of Nanjing Medical University, Nanjing, 210029 China; 4 Department of Cardiology, Wuhan Union Hospital, Wuhan, 430000 China

**Keywords:** Myocardial infarction, Myocardial infarction

## Abstract

Salidroside (SAL) is the major ingredient of *Rhodiola rosea*, and has been traditionally used in Chinese medicine for decades. Numerous studies have demonstrated the protective effects of SAL for myocardial ischemia. However, it is yet to be deciphered whether SAL has cardioprotective effects after myocardial infarction (MI) *in vivo*. In the present study, we established a mouse MI model via coronary artery ligation. The aim was to investigate whether SAL treatment could reduce mortality, improve cardiac function and attenuate myocardial remodeling in MI mice. Post-surgery, mice were randomly administered SAL or normal saline. After 21 days, SAL was found to significantly reduce mortality, improve cardiac function, reduce fibrosis and infarct size compared to normal saline. In addition, oral administration of SAL could attenuate myocardial inflammation and apoptosis and promote angiogenesis. SAL down-regulated the expression levels of TNF-α, TGF-β1, IL-1β, Bax and up-regulate the expression of Bcl-2, VEGF, Akt and eNOS. These results indicated that SAL could alleviate the pathological processes of myocardial remodeling in MI mice, and may be a potentially effective therapeutic approach for the management of clinical ischemic cardiovascular diseases.

## Introduction

Acute myocardial infarction (AMI) remains the most frequent cause of death worldwide. Nearly 20% of deaths are due to coronary heart disease^1^. Despite remarkable short-term improvements in post-MI survival over the past decades, the extent of left ventricular (LV) dysfunction has been associated with heart failure development and long-term mortality. Adverse LV myocardial remodeling following MI will lead to the thinning of the LV wall, LV dilation and systolic dysfunction. Myocardial remodeling is a significant cause of congestive heart failure^2–4^. Current treatments like Angiotensin-Converting Enzyme (ACE) inhibitors and beta-blockers can, at the best, slow the progression of heart failure and death. In addition, ACE inhibitors and beta-blockers are not suitable for patients with blood pressure below 90/60mmhg, and for patients with chronic respiratory diseases who have recurrent cough and dyspnea. Due to shortcomings of existing drugs, there is a critical need to find and develop alternative treatment strategies to prevent myocardial remodeling after MI and progression to heart failure.

Previous studies have demonstrated that in MI, continuous ischemia-induced cell death leads to irreversible tissue injury^5^. After AMI, cardiomyocytes undergo apoptosis and necrosis which results in infiltration of inflammatory cells and myocyte slippage^6^. This leads to the release of inflammatory factors such as tumor necrosis factor alpha (TNF-α), transforming growth factor beta 1 (TGF-β1) and interleukin 1 beta (IL-1β). It has been demonstrated that these inflammatory mediators contribute to myocardial fibroblast infiltration, inflammation and cell apoptosis in multiple animal disease models. In addition, they play crucial roles in the progression of myocardial remodeling after MI. Downregulation of these inflammatory factors can reduce inflammation, attenuate fibrosis and reduce cardiomyocytes apoptosis and necrosis^7–10^. Akt and vascular endothelial growth factor (VEGF) activate the PI3K/Akt pathway and downstream factors such as endothelial nitric oxide synthase (eNOS) to mediate both injury and repair. In addition, they can attenuate inflammatory response, reprogram cardiac fibroblasts into cardiomyocytes and inhibit myocardial cell apoptosis. Additionally these proteins promote angiogenesis and are involved in the formation of coronary collateral circulation, which has been demonstrated to limit MI and improve myocardial remodeling^11–14^.

Salidroside (SAL, p-hydroxyphenethyl-D-glucoside) is one of the major bioactive ingredients of *Rhodiola rosea*. It is a well-known herb and has been used traditionally to relieve high altitude sickness for hundreds of years^15–17^. Several studies have demonstrated its various biological properties, such as anti-inflammation, anti-fatigue, anti-stress, anti-hypoxia and anti-aging. It can reduce excessive blood lipids which results in improved blood circulation and enhanced immunity^18–20^. Recent studies have also demonstrated its multiple cardioprotective effects both *in vivo* and *in vitro*. It can reduce myocardial ischemia reperfusion injury, prevent atherosclerotic plaque formation and protect cardiomyocytes from oxidative injury^21–23^. However, whether SAL is a suitable treatment strategy for acute MI has not been demonstrated.

Mouse models have been useful in understanding MI pathogenesis^24^. In the present study, we established a mouse MI model by coronary artery ligation to investigate whether SAL could reduce mortality and improve cardiac function. We observed the protective effects of SAL on myocardial remodeling after MI and investigated its possible underlying mechanisms.

## Results

### SAL reduces mortality and improves cardiac function after MI

**Figure 1:**
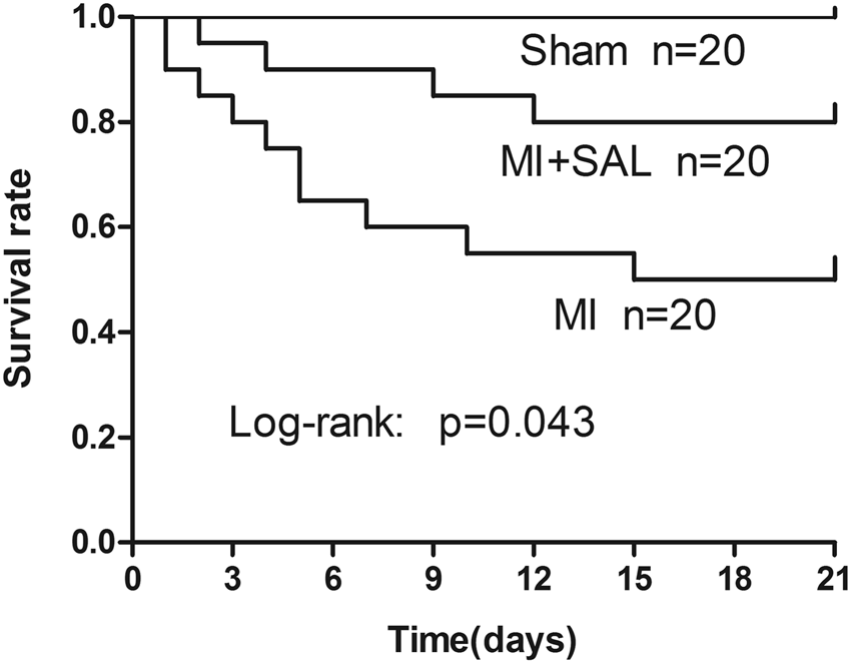
Survival curves for the three treatment groups 21 days post myocardial infarction (MI). Kaplan-Meier survival curves demonstrated that Salidroside (SAL) treated mice has lower mortality compared to MI mice (n = 20 per group, P < 0.05).

The survival rate of MI mice in the three treatment groups were measured for up to 3 weeks. There were no deaths in the sham- surgery group. The number of mice that survived in the SAL group was significantly higher compared to the MI group (80% vs 50%, P < 0.05; Fig. 1).

### SAL improves cardiac function after MI

**Figure 2:**
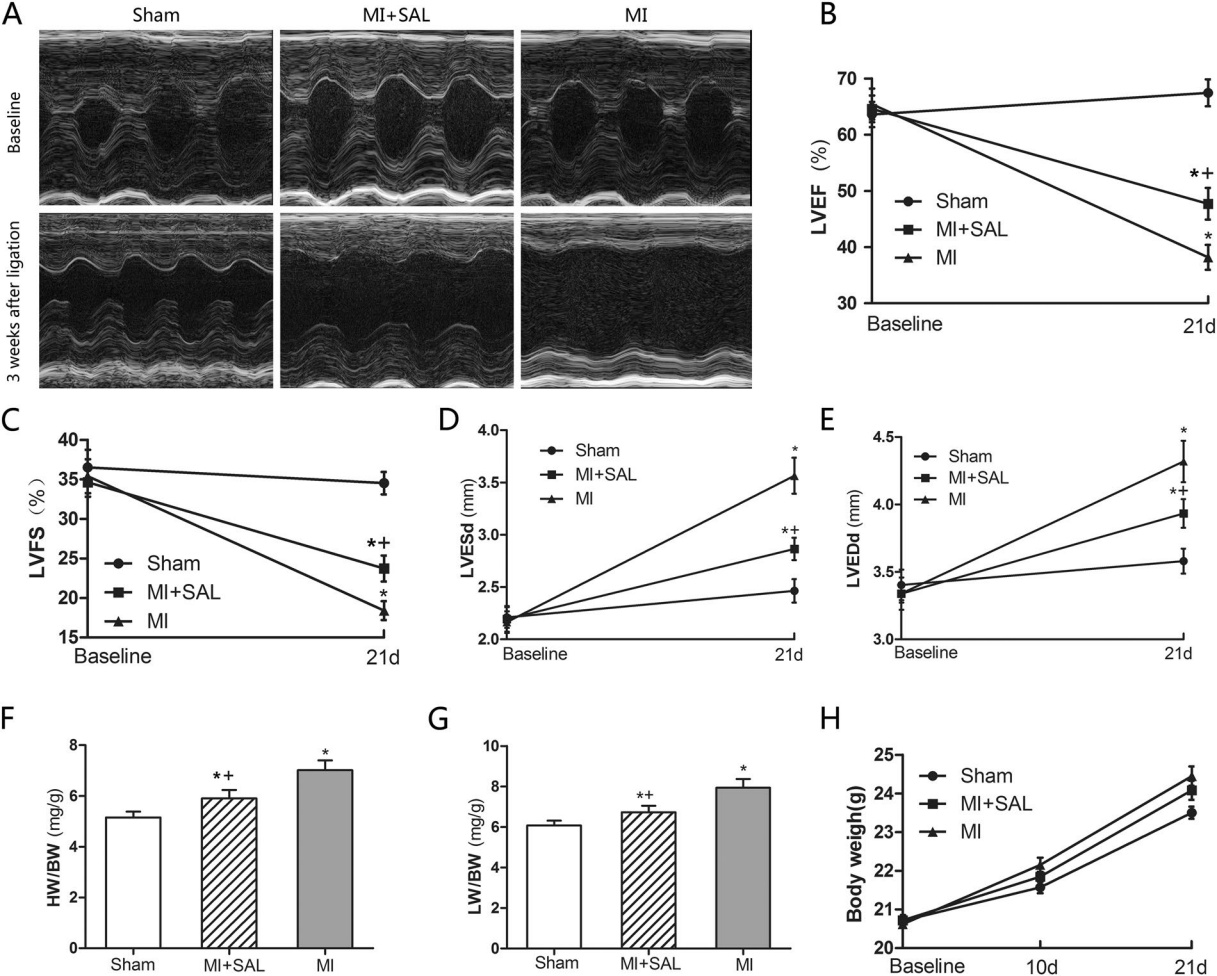
SAL improves cardiac remodeling and function after MI. (**A**) Representative images of M-mode echocardiographic in mice for the three treatment groups at baseline and 3 weeks after MI. (**B**) Analysis of the left ventricular ejection fraction (LVEF) at baseline and 21 days post-surgery. (**C**) Analysis of left ventricular fractional shortening (LVFS) at baseline and 21 days post-surgery. (**D**) Analysis of left ventricular end-systolic diameter (LVESd) at baseline and 21days post-surgery. (**E**) Analysis of left ventricular end-diastolic diameter (LVEDd) at baseline and 21 days post-surgery. (**F**) Analysis of heart weight/body weight ratio (HW/BW) at 21 days post-surgery. (**G**) Analysis of lung weight/body weight ratio (LW/BW) at 21 days post-surgery. (**H**) Body weigh curves for the three treatment groups. n = 10 per group.*P < 0.05 versus sham group, +P < 0.05 versus MI group.

To investigate the physio-pathological role of SAL in cardiac function, mice were examined by echocardiography. There were no significant difference between the three treatment groups at baseline. However, a significant myocardial thinning, chamber dilatation and systolic dysfunction, as characterized by increased LVEDd, increased LVESd, decreased LVEF and decreased left LVFS was observed in the MI group compared to the sham group (P < 0.05). However, LV dysfunction and dilatation were attenuated in the SAL treatment group compared to the MI group [**P < 0.05**, Fig. 2(A–E)]. For each mouse, the heart, lung and total body weight was measured to calculate the heart weight/body weight (HW/BW) ratio and lung weight/body weight ratio (LW/BW). The HW/BW and LW/BW were significantly lower in the SAL group compared to the MI group [**P < 0.05**, Fig. 2(F,G)]. These results suggest that SAL administration ameliorates cardiac remodeling and function after MI.

### SAL reduces cardiac infarct size and fibrosis after MI

**Figure 3:**
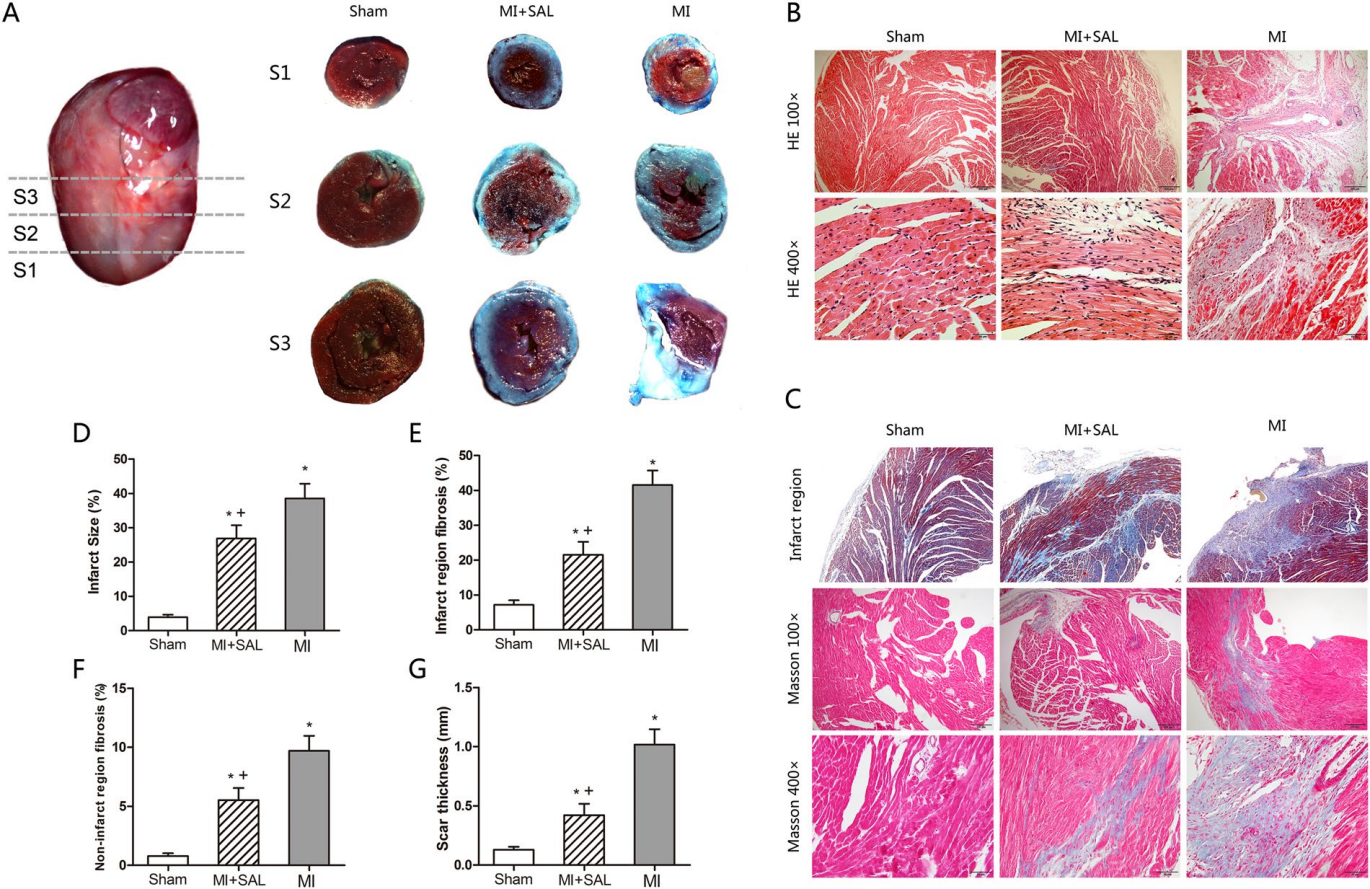
Effect of SAL on cardiac infarct size, morphology and fibrosis three weeks post MI. (**A**) Representative triphenyltetrazolium chloride (TTC) and Evans blue stained heart tissue of short-axis sections from the cardiac apex to base for the three treatment groups. Viable myocardium is shown in red and the infarct area shown in ash. (**B**) Representative hematein and eosin (HE) stained heart sections for the three treatment groups. (**C**) Representative Masson trichrome stained heart sections for the three treatment groups. (**D**) Graphical presentation of infarct size for the three treatment groups. (**E**) Graphical presentation of fibrosis size in infarct region for the three treatment groups. (**F**) Graphical presentation of fibrosis size in non-infarct region for the three treatment groups. (**G**) Graphical presentation of scar thickness for the three treatment groups. n = 6 per group. *P < 0.05 versus sham group, +P < 0.05 versus MI group.

To determine whether SAL administration attenuates cardiac injury, we measured the infarct size using TTC and Evans blue staining in mice for the three treatment groups three weeks after MI. Mice in the SAL group had a lower percentage of heart infarction compared to mice in the MI group [**P < 0.05**, Fig. 3(A,D)]. We then used HE and Masson trichrome to observe morphologic changes and fibrosis. Mice that were administered SAL had reduced myocardial fibrosis and collagen content both in non-infarct and infarct region compared to mice in the MI group. And mice treatment with SAL had a lower scar thickness compared to mice in the MI group [**P < 0.05**, Fig. 3(B,C,E,F)].

### SAL treatment reduced expression of inflammatory cytokines

**Figure 4:**
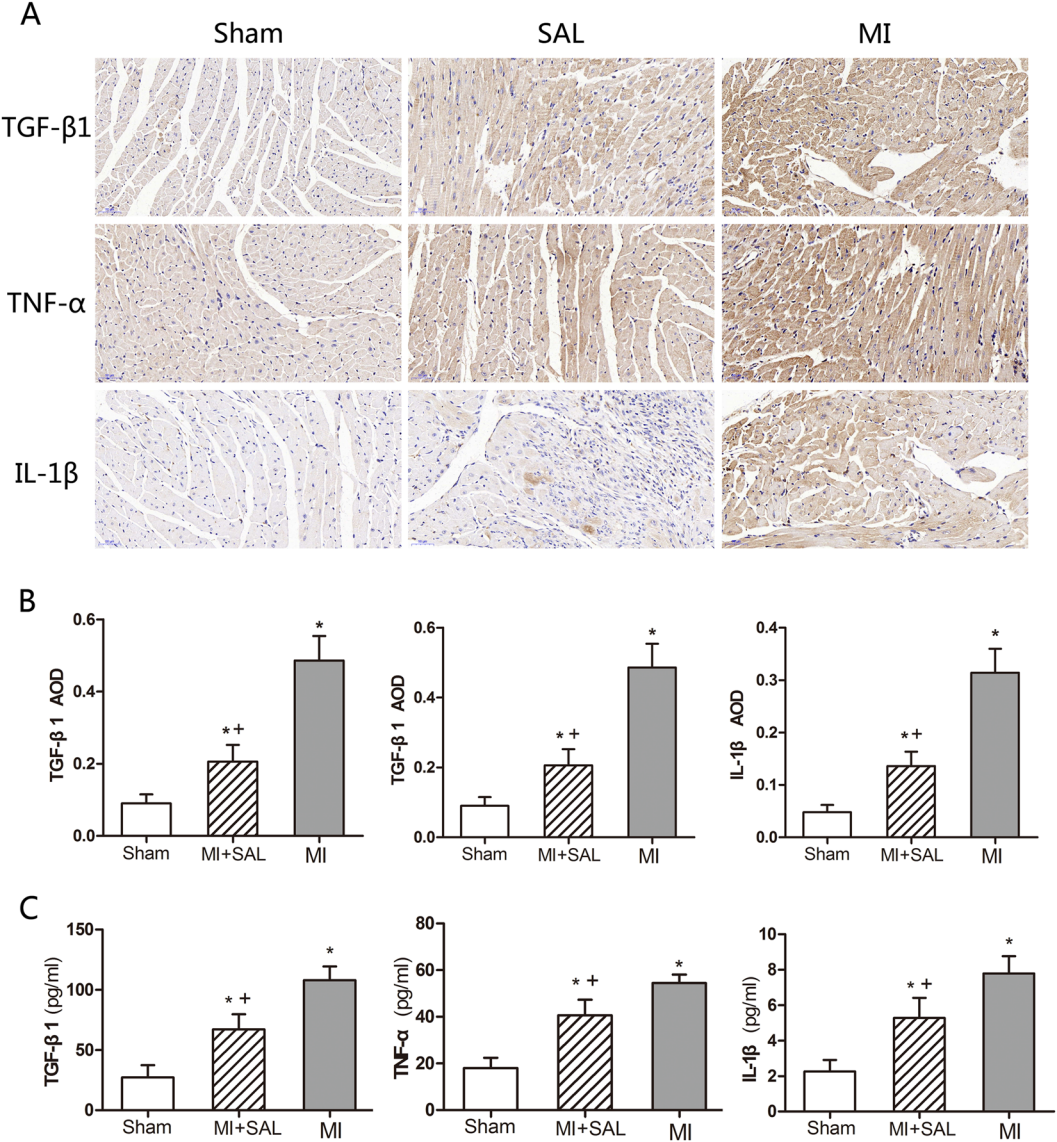
Effect of SAL on the expression of inflammation factors after MI. (**A**) Representative immunohistochemical staining images for transforming growth factor beta 1 (TGF-β1), tumor necrosis factor alpha (TNF-α), and interleukin 1 beta (IL-1β) for the three treatment groups after MI. (**B**) Average optical density (AOD) of immunohistochemical staining for the three treatment groups. (**C**) ELISA quantitative analysis for TGF-β1, TNF-α and IL-1β expression in left ventricular tissue lysates for the three treatment groups. n = 6 per group, *P < 0.05 versus sham group, +P < 0.05 versus MI group.

Immunohistochemistry staining and ELISA were used to measure the expression levels of inflammatory factors in cardiac tissue including both the non-infarct and scar region, i.e., TNF-α, TGF-β1, and IL-1β. MI increased the expression of inflammation cytokines. The expression levels of TNF-α, TGF-β1 and IL-1β were increased substantially in mice in the MI group compared to the sham group. However, TNF-α, TGF-β1 and IL-1β levels were significantly reduced in the SAL group compared to the MI group (P < 0.05), indicating that SAL treatment attenuated the expression of inflammatory cytokines [Fig. 4(A–C)].

### SAL treatment attenuates myocardial cell apoptosis after MI

**Figure 5:**
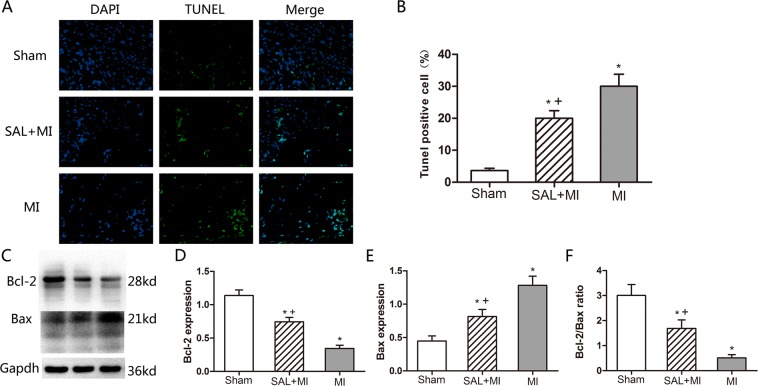
Effect of SAL treatment on myocardial cells apoptosis after MI. (**A**) Representative TUNEL staining images for the three treatment groups 21 days post MI. TUNEL positive cells are shown in green, DAPI positive cells are shown in blue. Cells that stained TUNEL and DAPI positive were considered apoptotic cardiomyocytes. (**B**) Analysis of cardiomyocyte apoptotic index for the three treatment groups. (**C**) Analysis of Bcl-2 and Bax expression by Western blotting. GAPDH was selected as the housekeeping gene. (**D**) Densitometry for Bcl-2 expression normalized to GAPDH. (E) Densitometry for Bax expression normalized to GAPDH. (**F**) The ratio of Bcl-2 and Bax for the three treatment groups. n = 6 per group. *P < 0.05 versus sham group, +P < 0.05 versus MI group.

The apoptosis levels of myocardial cells at the border regions were determined by TUNEL assays. Mice in the MI group had a significantly higher apoptotic index in ischemic myocardium tissues (P < 0.05) compared to mice in the sham group. However SAL treatment significantly decreased the apoptotic index compared to mice in the MI group [**P < 0.05**, Fig. 5(A,B)]. To determine the effects of SAL on the expression of apoptosis related proteins in cardiac tissue including both the non-infarct and scar region, we performed western blotting analysis for Bax and Bcl-2. Bcl-2 expression levels were significantly increased while Bax levels were significantly decreased in the SAL group compared to the MI group. The ratio of Bcl-2/Bax was significantly increased in the SAL group compared to the MI group [**P < 0.05**, Fig. 5(C–F)].

### SAL treatment promotes angiogenesis after MI

**Figure 6:**
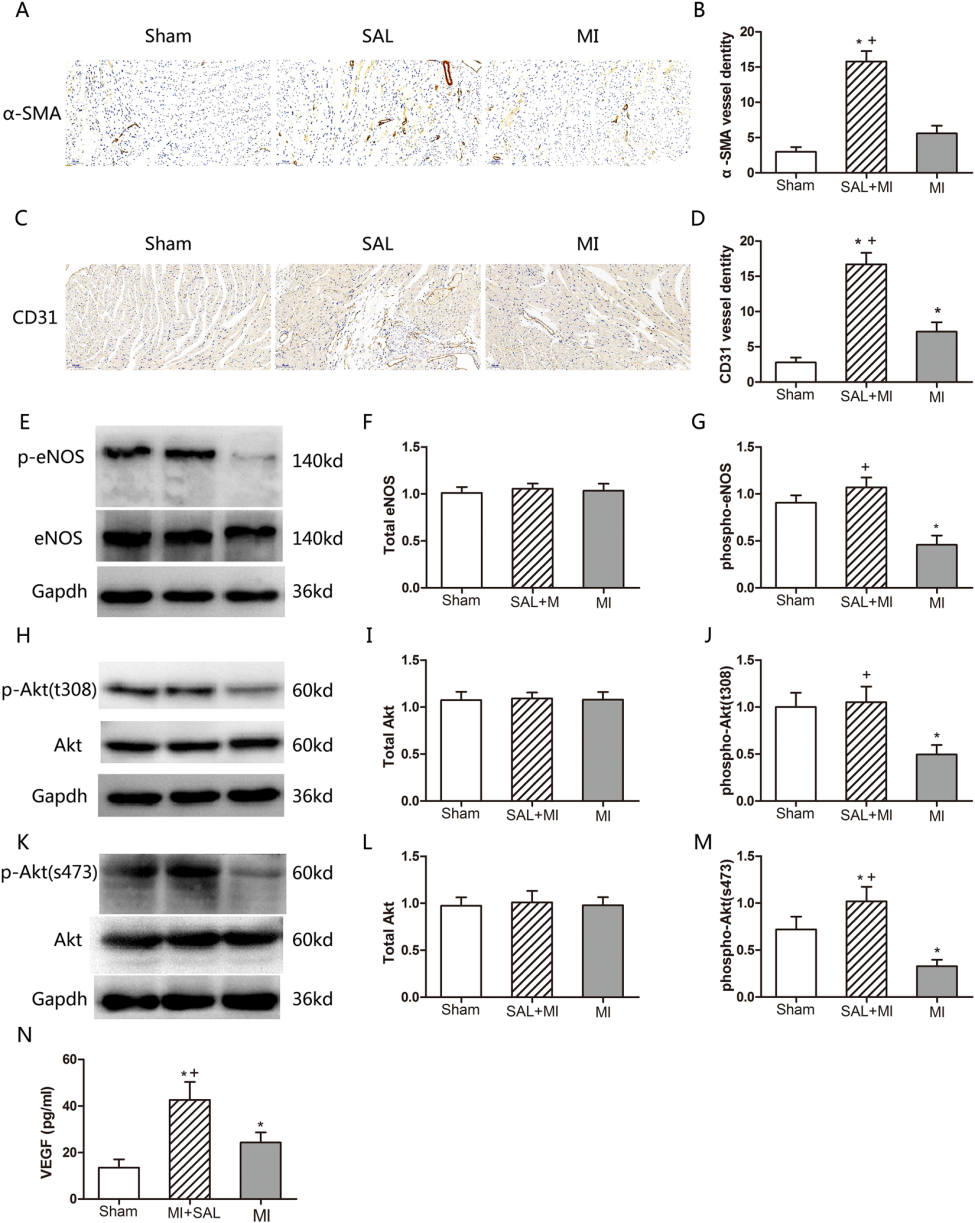
Effect of SAL treatment on angiogenesis after MI. (**A**) Representative α-SMA immunohistochemistry staining images for the three treatment groups. (**B**) Analysis α-SMA expression levels for the three treatment groups. (**C**) Representative CD31 immunohistochemistry staining images for the three treatment groups. (**D**) Analysis of CD31 expression levels for the three groups. (**E**) Analysis of eNOS and phospho-eNOS expression levels by Western blotting. GAPDH was selected as the house-keeping gene. (**F**) Densitometry for total eNOS expression normalized to GAPDH. (**G**) Densitometry for phospho-eNOS expression normalized to GAPDH. (**H**) Representative western blot images of total Akt and phospho-Akt (thr308) expression levels. (**I**) Densitometry for total Akt expression normalized to GAPDH. (**J**) Densitometry for phospho-Akt (thr308) expression normalized to GAPDH. (**K**) Representative western blot images of total Akt and phospho-Akt (ser473) expression levels. (**L**) Densitometry for total Akt expression normalized to GAPDH. (**M**) Densitometry for phospho-Akt (ser473) expression normalized to GAPDH. (**N**) Quantitative ELISA analysis of VEGF expression in left ventricular tissue lysates for the three groups. n = 6 per group.*P < 0.05 versus sham group, +P < 0.05 versus MI group.

The role of SAL in angiogenesis was determined using α-SMA and CD31 immunohistochemistry staining. SAL administration markedly increased microvessel formation at the border zones compared to mice in the MI group [**P < 0.05**, Fig. 6(A–D)]. Total Akt and eNOS expression levels in cardiac tissue including both the non-infarct and scar region were similar for all groups. However, after SAL treatment, phosphorylated Akt (both thr308 and ser473) and phosphorylated eNOS protein levels were significantly higher compared to mice in the MI group [**P < 0.05**, Fig. 6(E–M)]. Similarly, VEGF expression levels were significantly higher in the SAL group compared to the MI group [**P < 0.05**, Fig. 6(K)]. These results demonstrate that SAL had ability to promote angiogenesis after MI. The underlying mechanisms might be the activation of the VEGF-Akt-eNOS pathway.

## Discussion

In this study we found that compared to mice in the MI group, mice treated with SAL had significantly reduced mortality, improved systolic cardiac function and reduced fibrosis and infarct size after MI. SAL administration attenuated myocardial inflammation, apoptosis and promoted angiogenesis, and alleviated the pathological process of myocardial remodeling induced by MI. The underlying mechanisms may be via the down-regulation of TNF-α, TGF-β1, IL-1β, Bax, and the up-regulation of Bcl-2, VEGF, Akt and eNOS expression to attenuate myocardial ischemia, enhance neovascularization and cardiac repair. These results demonstrated that SAL could be an effective drug against myocardial remodeling induced by MI.

Previous studies have demonstrated that MI causes ischemia-induced cell death leading to irreversible tissue injury, inflammatory cell infiltration and release of inflammatory factors such as TNF-α, TGF-β1, IL-1β^25^. These inflammatory cytokines can regulate gene expression in multiple cell types to result in diverse biological effects. After tissue damage, multiple inflammatory factors can trigger phosphorylations on key proteins resulting in nuclear translocation of transcription factors. These inflammatory cytokines play a crucial role in myocardial fibrosis, cardiomyocyte apoptosis and the pathological process of myocardial remodeling. In addition, myocardial fibroblast infiltration enlarges the fibrotic region leading to LV dilation and dysfunction, resulting in irreversible HF^26–28^. Consistent with previous studies, we found that after MI, the expression of inflammatory factors were significantly increased in myocardium tissue, and resulted in myocardial remodeling. In addition, previous studies have demonstrated that SAL has anti-fibrotic and anti-inflammatory activities both *in vitro* and in *vivo*^29,30^. Similarly, our results demonstrated that SAL administration could reduce the upregulation of TNF-α, TGF-β1 and IL-1β after MI, and significantly reduce fibrosis. This demonstrated that SAL had anti- fibrotic and anti-inflammatory effects after MI.

Apoptosis is a key pathological feature in the progression of MI and myocardial remodeling^31,32^. Previous studies have demonstrated that ischemic-induced necrosis and apoptosis leads to cardiomyocyte loss and autophagic cardiomyocyte death after MI^33,34^. Bcl-2 and Bax are critical apoptosis factors induced after MI^35^. Bcl-2 protein family is critical for regulating apoptosis and cell survival^36^. Bax is a pro-apoptotic protein. Bcl-2 can inhibit apoptosis by forming heterodimers with Bax to inhibit its activity. In addition, the Bcl-2 protein family regulates the mitochondria-mediated intrinsic pathway to inhibit apoptosis. In AMI, inflammatory cytokines released by the injured myocardium mentioned, i.e., IL-1β and TNF-α can also induce myocardial apoptosis by activating Bax and BCL family members. This in turn aggravates myocardial remodeling^27,37^.

The present study demonstrated that SAL has anti-apoptotic effects both *in vivo* and *in vitro*^38,39^. We demonstrated that SAL treatment could reduce apoptosis in mice after MI. Using TUNEL assays, the number of apoptotic cells were significantly reduced at the border region of the infarcted myocardium after SAL treatment. In addition, we observed increased Bcl-2 expression and decreased Bax expression after SAL treatment. The increased Bcl-2/Bax ratio attenuated myocytes apoptosis under ischemic conditions. Our findings indicated that SAL has anti-apoptotic effects during myocardial ischemic injury. However, the detail mechanisms of this process needs to be deciphered.

Several studies have shown that activation of the VEGF-Akt- eNOS signaling pathway is associated with cardioprotective effects after MI^40,41^. VEGF is an endothelial specific mitogen and survival factor. It is one of the most potent angiogenic factors and it is a key factor for both angiogenesis and vasculogenesis^13^. VEGF activates PI3K/Akt signaling pathway and up-regulates eNOS expression. eNOS can be activated by phosphorylation at serine1177 residue to generate nitric oxide, which induces endothelial cell migration and angiogenesis. This sequentially mediates both injury and repair^42–44^. This process is associated with the formation of coronary collateral circulation. We observed that VEGF levels were increased after SAL administration in MI mice. In addition, we observed that SAL administration stimulated Akt and eNOS phosphorylation. p-Akt and p-eNOS expression levels were significantly increased compared to mice in the MI group. Circulating endothelial cells (CD31) have been reported to participate in blood vessel formation during both physiological and pathological processes, such as inflammation, wound healing, cardiovascular diseases, and cancer^45^. α-SMA is a maker used to measure vessel density^46^. After SAL administration, we observed increased CD31 and α-SMA positive vessel density in myocardial tissue suggesting the initiation of angiogenesis. All these factors and proteins limits MI and improves myocardial remodeling. Based on these results, we hypothesized that SAL could activate the VEGF-Akt-eNOS signaling pathway, thus promoting angiogenesis and the formation of the coronary collateral circulation. However, this is only a hypothesis, with no direct evidence for their intrinsic connection.

To the best of our knowledge, our study is the first to report that SAL could reduce mortality and improve cardiac function in MI mice. Moreover, SAL was effective in ameliorating myocardial remodeling after MI partially via the partial inhibition of myocardial apoptosis, inflammation, fibrosis and promoting angiogenesis.

There were several limitations in our study that needs to be stated. First, only focused pathways and mechanisms were investigated. Specific pathways and mechanisms induced by SAL needs to be deciphered. Second, whether combinations of SAL with other medications like ACE inhibitors and beta-blockers could result in better therapeutic efficacy needs to be investigated. Thirdly, the identity of cell undergoing apoptosis was not determined. Fourthly, it is not known how a circulating cytokine results in altered expression levels in cardiomyocytes. Lastly, the number of mice for each treatment group was small. Future detailed studies are required to fully understand the protective effects of SAL against ischemic heart diseases.

In conclusion, we demonstrated that SAL treatment can efficiently reduce mortality, improve systolic cardiac function and attenuate the pathological process of heart remodeling induced by MI. SAL therapy may be a potential therapeutic strategy for the management of clinical ischemic cardiovascular diseases.

## Materials and Methods

### Animals and ethics statement

One hundred and twenty C57BL/6 J male mice weighing 20–25 g (aged 7–8 weeks) were purchased from the Animal Center of Xuzhou Medical University. Mice were housed in specific pathogen free environment with a relative humidity of 50 ± 5% at 23 ± 2 °C with 12 h light and dark cycles. Mice had free access to food and water. All studies involving animals were performed according to the guidelines for the Care and Use of Laboratory Animals (Laboratory Animal Center of Xuzhou Medical University). All animal experiment protocols were approved by the Ethics Review committee for Lab Animal Use at Xuzhou Medical University.

### Mouse model for myocardial infarction and experimental design

Briefly, mice were anesthetized by intraperitoneal injection of pentobarbital sodium (50 mg/kg). After anesthesia had taken effected, mice were placed on a heating pad to maintain normothermia (about 35°C). Electrocardiogram (ECG), heart rate and respiratory rates were continuously monitored. Mice were then intubated and ventilated to perform left thoracotomy to expose the heart. MI was induced by permanent ligation of the LAD coronary artery using an 8–0 polypropylene suture passed about 2–3 mm from the inferior margin of the left auricle. Ischemia was confirmed by ECG ST-segment elevation and myocardial blanching. Sham surgeries were performed using an identical procedure except no sutures on the coronary artery were performed. Subsequently the thorax was closed and mice were intramuscular injected of penicillin to prevent infection. Morphin was used as analgesic for post-operative care (2 mg/kg, subcutaneous injection, q4h). After restoration of spontaneous breathing, endotracheal intubation was removed and the mice were placed on electric blankets until revival^47^. Mice with LAD ligation were randomly divided into two groups: the SAL group (n = 20) and the MI group (n = 20). Mice that had undergone the sham procedure were assigned to the Sham group (n = 20). Mice in the SAL group were administered with 200 mg/kg/day SAL intragastrically. Medication for the first time was 1 hr after MI. Mice in the Sham and MI groups were treated with saline intragastrically. SAL was purchased from Nantong Feiyu Biological Technology Co. LTD (Product batch number: FY12890409, Nantong, Jiangsu, China, purity >98%). The study was performed on 2 batches of 60 mice. The first 60 mice were sacrificed using carbon dioxide (CO_2_) at 21 days post-surgery which were used to obverse mortality, obtain ultrasound data, histopathology, immuno-histochemistry and TUNEL staining. The second 60 mice were sacrificed at the 7th day after surgery to obtain protein lysates. These protein lysates were used to perform enzyme-linked immunosorbent assay (ELISA) and Western Blot experiments.

### Echocardiography measurement

Mice were anesthetized with 1–2% isoflurane vapor in a 1:1 air: oxygen mixture via a nose cone and positioned on a heating pad to maintain normothermia. Transthoracic echocardiography was evaluated using a Vevo2100 imaging system (VisualSonics Inc, Toronto, ON, Canada) with a 30 MHz central frequency scan head. The ECG, heart rate and respiratory rate were continuously monitored. Two-dimensional echocardiographic views of the midventricular short axis were obtained. From M-mode tracing, left ventricular end-diastolic diameter (LVEDd), left ventricular end-systolic diameter (LVESd), left ventricular posterior wall end-diastolic thickness, left ventricular posterior wall end-systolic thickness, interventricular septum end-diastolic thickness and interventricular septum end-systolic thickness were measured. Left ventricular end-diastolic volume, left ventricular end-systolic volume, left ventricular ejection fraction (LVEF) and left ventricular fractional shortening (LVFS) were calculated using the formulas described in detail in our previous publication^47^.

### Measurement of myocardial infarct size

21 days post-surgery, infarct size was determined using Evans blue and TTC staining, as previously described^47^. Briefly, mice were intraperitoneally anesthetized using pentobarbital sodium (50 mg/kg). One ml of Evans blue dye (0.1 g/ml; BioSharp, China) was injected into the abdominal aorta. The heart was then quickly removed and weighed. After 30 minutes at −20 °C, the heart was cut into 4 or 5 transverse slices across the long axis. Each slice had a thickness of 1–2 mm. The slices were incubated in citrate buffer solution (ph = 7.4) for 30 min at 37 °C with 1% triphenyltetrazolium chloride (TTC, Amresco, USA) and then placed overnight in 4% paraformaldehyde. The infarct area was pale white, while the non-infarct area was red. Each slice was photographed and analyzed. The percentage of heart infarct size was calculated using computerized planimetry (Image J, version 1.44, NIH, Bethesda, MD). We calculated the percentage of infarct size for each slice and then averaged the slices.

### Histopathology

Twenty-one days post-surgery, all mice were humanely sacrificed. Hearts were harvested, washed in PBS and placed overnight in 10% formaldehyde and then embedded in paraffin. Each heart was cut into sections of 4–5 μm and stained with hematein and eosin (H&E) to evaluate morphological changes. Masson trichrome staining was used to observe the scar thickness and extent of cardiac fibrosis^47^. Each section was photographed using an imaging microscope (Nikon, Japan). The average scar thickness was measured from 3 sites of LV scar. We measured the relative LV fibrosis area (fibrosis area divided by myocardium area). We selected the slide which contained the largest infarct size to determine the data. We selected the same slide from each group. For each slide, 5 separate fields were examined randomly and digitized by microscopy at a magnification of 100×. All five fields were in the same position in all three groups.

### Immunohistochemistry staining

Paraffin embedded myocardial samples were dewaxed and rehydrated in xylene and ethyl alcohol followed by incubation in 0.3% methanol/H_2_O_2_ to block endogenous peroxidases. After antigen retrieval was performed in citrate buffer (pH 6.0, 95–100 °C), the sections were incubated overnight with the following primary antibodies (α-SMA,CD31, 1:200, Cell Signaling Technology, USA; TGF-β, TNF-α, IL-1β, Abcam, USA) at 4 °C. Two-step technique (SuperPictureTM3rd Gen IHC Detection kit; Invitrogen, CA, USA) was used for visualization, using 3, 3′-diaminobenzidine (DAB, 0.1 mg/ml, 0.02% H_2_O_2_, Vector Laboratories, Burlingame, USA) as the chromogen. Sections were counterstained with hematoxylin. We selected the same slide which contained the largest infarct size for each group to ensure the comparability of the three groups. For each slide, 5 separate fields were examined randomly and digitized by microscopy at a magnification of 400×. All five fields were in the same position in all three groups. Image-Pro Plus software (Media Cybernetics, Rockville, USA) was used to determine the area of α-SMA, CD31, TGF-β, TNF-α and IL-1β positive staining.

### Measurement of apoptotic cells by TUNEL assays

The number and distribution of apoptotic cells were measured using an apoptosis *in situ* Cell Death Detection Kit (Biouniquer, Nanjing, China) according to the instructions provided by the manufacturer^47^. All slices were stained with DAPI (1 μg/ml; Sigma, St Louis, USA) for the assessment of nuclear morphology. FITC-labeled TUNEL-positive cells were imaged using a fluorescent microscopy at 400 × magnification (Nikon, Japan). We selected the same slide which contained the largest infarct size for each group to ensure the comparability of the three groups. Five areas were randomly selected from each section. All five fields were in the same position in all three groups. FITC-labeled TUNEL-positive cells were counted using the Image-Pro Plus software (Media Cybernetics, Rockville, USA). Apoptotic index = apoptosis cell number/1000 cells*100%.

### Western blot analysis

Protein expression was measured by western blot^47^. Briefly, after the mice were sacrificed, hearts were harvested. The Atrium and right ventricle were then removed. The protein was obtained from the left ventricular myocardial tissue including both the non-infarcted and scar region. After centrifugation, samples were sonicated and heat denatured (95–100 °C for 5 min with SDS loading buffer). Protein concentration was determined using the BCA protein Assay Kit (Beyotime, China). A total of 20ug protein lysates were electrophoresed and separated using a 6%-12% SDS-PAGE and then transferred onto nitrocellulose membranes (Bio-Rad, Hercules, USA). The membranes were then blocked with 5% skim milk at 25 °C for an hour and then incubated over night at 4 °C with the following primary antibodies; eNOS (1:1000; Cell Signaling Technology, USA), phospho-eNOS (1:200; Santa Cruz Biotechnology, Santa Cruz, USA), Akt (1:1000; Cell Signaling Technology, USA), phospho-Akt (thr308) (1:1000; Cell Signaling Technology, USA), phospho-Akt (ser473) (1:1000; Cell Signaling Technology, USA), Bcl-2 (1:800; Bioworld, USA), Bax (1:800; Bioworld, USA), GAPDH (1:1000; Cell Signaling Technology, USA). Afterwards the membranes were incubated for 1 hour at 25 °C with HRP-conjugated secondary antibody (1:500; Santa Cruz Biotechnology, Santa Cruz, USA). The antigen–antibody complexes were detected using a SuperSignal ECL kit (Thermo, USA) in a Western blotting detection system (Bio-Rad, CA, USA). Results were expressed as density values normalized to GAPDH levels.

### ELISA analysis

The ELISA kit (Bio-Swamp, Shanghai, China) was used to determine TGF-β1, TNF-α, IL-1β and VEGF levels from left ventricle samples^47^. In brief, after the mice were sacrificed, hearts were harvested. The Atrium and right ventricle were then removed. The protein was obtained from the left ventricular myocardial tissue including both the non-infarct area and scar region. 20 mg myocardial tissue samples were homogenized in 200 ul of 1 × PBS (pH = 7.4), then stored overnight at −20 °C. After two freeze-thaw cycles to dissociate the cell membranes, the homogenates were centrifuged at 5000 *g* for 10 minutes. Samples were assayed immediately following the procedure recommended by the manufacturer.

### Statistical analysis

SPSS 18.0 or GraphPad Prism 5 were used to perform statistical analyses. Results were expressed as mean ± standard error of mean. One-way Anova analysis was used to compare data among the three groups. Comparisons between two groups were performed using One-way post-hoc test. Data that did not conform to normal distribution were analyzed using the Kruskall-Wallis test. Kaplan-Meier curve survival analysis and comparisons using log-rank test was performed to determine overall survival. P < 0.05 was considered statistically significant.

## Data Availability

Data generated from this study is available upon reasonable request from Dr. Bing Han (Department of Cardiology, XuZhou Central Hospital, Xuzhou Clinical School of Nanjing Medical University, XuZhou Institute of Cardiovascular disease).
